# Analysis of the Effectiveness of Lornoxicam and Flurbiprofen on Management of Pain and Sequelae Following Third Molar Surgery: A Randomized, Controlled, Clinical Trial

**DOI:** 10.3390/jcm8030325

**Published:** 2019-03-07

**Authors:** Gaetano Isola, Angela Alibrandi, Eugenio Pedullà, Vincenzo Grassia, Sebastiano Ferlito, Letizia Perillo, Ernesto Rapisarda

**Affiliations:** 1 Department of General Surgery and Surgical-Medical Specialties, School of Dentistry, University of Catania, 95124 Catania, Italy; eugeniopedulla@gmail.com (E.P.); sferlito@unict.it (S.F.); erapisa@unict.it (E.R.); 2 Department of Economical, Business and Environmental Sciences and Quantitative Methods, University of Messina, 98122 Messina, Italy; aalibrandi@unime.it (A.A.); 3 Department of Surgical and Dental Specialties, University of Campania “Luigi Vanvitelli”, 80138 Naples, Italy; grassiavincenzo@libero.it (V.G.); letizia.perillo@unicampania.it (L.P.)

**Keywords:** third molar surgery, Flurbiprofen, Lornoxicam, pain, swelling, trismus, randomized clinical trial

## Abstract

The aim of this study was to analyze the effectiveness of Lornoxicam and Flurbiprofen in reducing perioperative sequelae after impacted mandibular third molar surgery. Ninety-one patients who needed surgical extraction of an impacted mandibular third molar were selected for the study. All subjects were randomly allocated to receive one of the following treatments twice a day for 5 days after surgery: placebo (*n* = 29), Flurbiprofen (*n* = 31), or Lornoxicam (*n* = 31). The primary outcome was postoperative pain, evaluated using the visual analogue scale (VAS) score at 30 min, 2, 6, 12, 24, 48 h, 7 and 10 days following surgery. The secondary outcomes chosen were changes in postoperative swelling and maximum mouth opening values compared to preoperative ones. Compared to placebo, treatment with Flurbiprofen and Lornoxicam was characterised by an improvement in the primary outcome. Moreover, the treatment with Lornoxicam presented significantly lower median pain scores at 2 h (*p* < 0.001) and at 6 h (*p* = 0.016) compared to Flurbiprofen and at 2 h (*p* < 0.001), 6 h (*p* = 0.01), and at 24 h (*p* = 0.018) after surgery compared with placebo. Swelling and maximum mouth opening values were not significantly different between the groups at each follow-up session. This trial demonstrated that treatment with Lornoxicam showed a decrease in the incidence and severity of pain in the first postoperative phase following third molar surgery compared to Flurbiprofen and placebo.

## 1. Introduction

Mandibular third molar surgery is one of the most commonly performed oral surgery procedures and is associated with a wide range of symptoms such as pain, swelling, and other important oral inflammatory sequelae [1]. Following the surgical extraction of the third molar, a number of inflammatory mediators are released including leukotrienes, prostaglandins, and platelet activating factors [2]. The production of these inflammatory mediators results in an increase in the vasodilatation and vascular permeability of the surgical site, leading to peripheral edema and local tissue alterations [3].

The correct management of these inflammatory sequelae, mediators, and pain forms the basis of successful postoperative management [4]. Many strategies have been developed to reduce clinical signs and symptoms following third molar surgery, including the use of pharmacological therapy to inhibit the release of the inflammatory mediators responsible for this acute response [5].

Non-steroidal anti-inflammatory drugs (NSAIDs) and corticosteroids are the most widely used drugs, as other adjuvant local agents, in post-surgical oral and periodontal therapy due to their anti-inflammatory and analgesic properties [6,7]. Among the main NSAIDs, the most important are those which inhibit the release of prostaglandin E2 (PGE2) and a decrease the release of serotonin by the nervous system, producing a relatively strong reduction in inflammation at the systemic and topic level [8].

It has been previously shown that NSAIDs mainly exert their anti-inflammatory action by inhibiting cyclooxygenases (COX)-1 and COX-2. However, as the inhibition of COX-1 is associated with a lack of gastric protection [9], a new class of NSAIDs with selective anti-inflammatory activity has been developed in recent decades, called -oxicam. In fact, with a rapid onset of action and with a short half-life, it provides excellent anti-inflammatory efficacy with almost no influence on the gastrointestinal tract [9]. However, although the efficacy of -oxicam has been demonstrated extensively in patients undergoing general surgery [10], there is currently no robust evidence indicating the same efficacy of -oxicam NSAIDs for the treatment of the acute postoperative discomfort phase after the surgical extraction of impacted third molars [11,12].

A recent randomized controlled trial studied the effects of a new and potent NSAID of the -oxicam class with anti-inflammatory and analgesic effects; Lornoxicam was used for the management of postoperative pain in patients undergoing third molar surgery [13]. The results of that pilot study showed efficacy in the management of the acute postoperative pain phase for a pre-emptive dose of 16 mg of Lornoxicam compared to the placebo [13]. Moreover, a similar clinical study which analysed the effectiveness of 16 mg Lornoxicam compared with ibuprofen showed that Lornoxicam was equally effective to ibuprofen for postoperative pain discomfort after the surgical extraction of impacted mandibular third molars [14]. These preliminary results were validated by a recent randomized, double-blind clinical trial which assessed the pain-relieving activity of another -oxicam, Tenoxicam [15]. When used, Tenoxicam showed good results in providing effective long-lasting analgesia at relatively low doses in the management of acute postoperative pain following third molar surgery.

On the basis of these encouraging pivotal results, the objective of this study was to evaluate the efficacy of Lornoxicam versus a commonly used anti-inflammatory drug (Flurbiprofen) and a placebo in the management of the postoperative discomfort after the surgical removal of mandibular third molars. The null hypothesis was that there would be no difference between the three protocols analysed.

## 2. Materials and Methods

### 2.1. Study Design

The study was designed as a single-center, randomized, triple-blind controlled clinical trial. The study was performed in accordance with the Declaration of Helsinki on medical research, as revised in 2016. Ethical approval was obtained at the beginning of the study from the Institutional Review Board of the University of Messina (#12–16). Written informed consent was obtained from each patient, all of whom were informed about the study protocol (the randomized, blinded and controlled clinical trial study design, the nature of the surgical procedures, and the characteristics of the drugs used) and possible risks of the study (short- and long-term injuries linked to the surgical procedure and the possible allergic sequelae or complications linked to the drugs used) before any procedures were performed.

All subjects were consecutively and randomly recruited from normal healthy subjects (according to the American Society of Anaesthesiology (ASA) classification, ASA I), aged ≥18 years, who required the surgical removal of an impacted third molar in the mandible. The enrolment of patients was performed between May 2016 and July 2018 at the Unit of Oral Surgery in the Department of Odontostomatology of the University of Messina, Messina, Italy.

**Figure 1:**
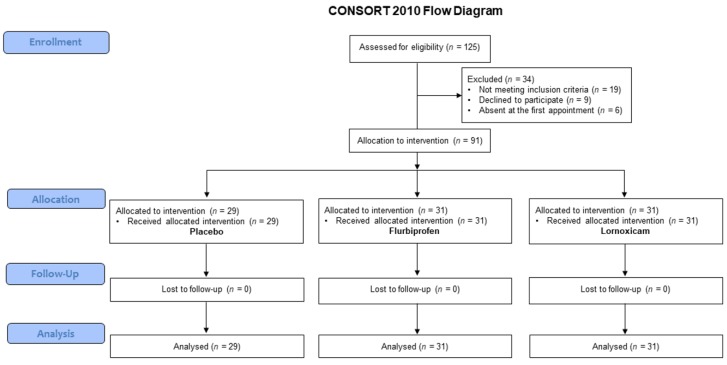
Flowchart of patient recruitment into the study groups.

The inclusion criteria were as follows: (1) aged between 18 and 32 years; (2) good general health; (3) the presence of one impacted third molar in the mandible with a class II position, type B impaction [16]; (4) absence of pericoronitis or signs of inflammation during the last 30 days. Panoramic radiographs were used to determine tooth position. The exclusion criteria were as follows: (1) the presence of any systemic disease; (2) consumption of oral contraceptives or other medications; (3) consumption of any immunosuppressive or anti-inflammatory drugs during the 3 months prior to the study; (4) status of pregnancy or lactation; (5) previous history of excessive drinking; (6) allergy to local anesthetic; (7) smoking habit. The study was performed according to the CONSORT (Consolidated Standards of Reporting Trials) guidelines (Figure 1; Table S1) [17]. Patients were excluded if they did not complete the study or if they did not specifically follow the study protocol. Moreover, patients were excluded if any surgical time went over 40 min.

### 2.2. Sample Size Analysis and Procedures

The sample size calculation was performed taking into account the identification of the three groups, with an effect size of 0.40, α = 0.050, and with a power level of 0.80 for pain, which represented the primary variable selected for the analysis. The primary variable ‘pain’ presented a difference between groups of 0.62 (mean) and a standard deviation (SD) of 0.73. After an evaluation of these values, it was calculated that a minimum number of 29 patients in each group was necessary.

An inter-examiner reliability test was performed for the study, which showed an agreement of 84.6% (κ = 0.59) for the primary outcome (pain). Moreover, the intra-examiner agreement was evaluated by the measurement of Cohen’s kappa coefficient; this was κ = 0.834, indicating a high degree of reliability. The kappa coefficient was also calculated for the measurements taken at each follow-up session and an acceptable degree of reliability was established for every examination (intra-class correlation coefficient t = 0.764).

In the first stage of the study, 125 patients were recruited (62 male, 63 female) from all patients who were referred to the Department of Odontostomatology of the University of Messina were recruited. After evaluation against the study criteria, 34 subjects (15 male, 19 female) were excluded. These subjects did not meet the eligibility criteria (*n* = 19), refused to participate in the study (*n* = 9), or were absent at the baseline (*n* = 6). Therefore, a final number of 91 patients were included in the analysis (Figure 1).

All patients attended an initial preoperative screening consultation, which was performed by the same experienced clinician, who was blinded to the test medication administered. Patient data including age, sex, systemic diseases, coagulation and glycaemic parameters, and periodontal status were recorded preoperatively. The panoramic radiographs obtained prior to enrolment were examined to re-evaluate the tooth position, the degree of tooth impaction, and the degree of tooth/root formation of each third molar.

Each enrolled subject was assigned to one of three groups in accordance with the postoperative medication received: the placebo group (*n* = 29) was given a placebo capsule (sugar pill, Sucratol-placebo capsules) twice a day for 5 days; the Flurbiprofen group (*n* = 31) were given 200 mg Flurbiprofen twice a day for 5 days (Froben 100mg, BGP Products S.R.L., Italy, 2 capsules); the Lornoxicam group (*n* = 31) were given 16mg Lornoxicam capsules (Taigalor 8 mg, Nycomed Italy S.r.l., Italy, 2 capsules) twice a day for 5 days. Before the procedure, each patient received 1 g Amoxicillin with clavulanic acid as preoperative prophylactic therapy, 1 h before surgery (Augmentin; GlaxoSmithKline, Milan, Italy).

All patients were allowed to extend the therapy up to 8 days if they presented a postoperative visual analogue scale (VAS) score for pain of ≥4 at 5 days after surgery [18]. No postoperative antibiotics were prescribed, but 0.12% chlorhexidine mouthwash three times a day for 7 days was prescribed for all enrolled subjects.

### 2.3. Randomization

The assignment of each patient to a study group was determined by means of a randomization technique using sealed and numbered envelopes; details of the sequence were concealed from all clinicians who participated in the study. An operator not involved in the subsequent experimentation generated a random allocation sequence, in a 1:1:1 ratio, for the distribution of the patients to one of the three study groups, performed with a permuted block design using a computer generator.

Before the start of each treatment, an operator who was not involved in the subsequent phases of the study or the data processing conducted the assignment of each sealed envelope (which contained the type of treatment and the patient’s name and date of birth) to the therapist who subsequently prescribed postoperative therapy. The same operator who performed the statistical assignment phase was blinded to all patient clinical data and to the following analysis and evaluation of the data. For the codification of the single groups, it was decided, before the study, to assign the letter ‘A’ to the placebo treatment, the letter ‘B’ to the Flurbiprofen treatment, and the letter ‘C’ to the Lornoxicam treatment. Each envelope was used for each treatment assignment. In this phase, the type of treatment for each patient, useful for the subsequent statistical analysis, was registered. The patient, clinician, surgeon, and statistician who participated in the subsequent follow-up session were all blinded to the treatment data.

### 2.4. Treatment

All surgical procedures were performed by the same clinician in order to avoid any possible bias with regard to surgeon variability. Each enrolled subject underwent the same surgical extraction procedure, performed under similar clinical conditions. A local anesthetic technique was employed that included inferior alveolar nerve blocks using Mepivacaine 2% with Epinephrine 1:100000 (Molteni Farmaceutici S.p.A., Scandicci, Italy). The total amount of local anesthetic used for the operation was recorded for each patient, by summing the number of dental cartridges used.

The same mucoperiosteal flap with subsequent osteotomy (useful for accessing the tooth) was performed in all patients. The bone was removed with a round bur in a straight hand-piece under continuous saline solution irrigation. In all subjects, tooth sectioning and removal of the third molar was performed, following which granulation tissue was removed from the alveolar cavity. The surgical wound was closed using a 4–0 resorbable suture (Coated Vicryl (polyglactin 910); Ethicon, Somerville, N.J., USA).

Immediately after surgery, postoperative therapy was carefully explained to each patient. They were instructed to follow a liquid and cold diet for the first 24 h. Patients were also motivated by oral hygiene and the possible symptomatology resulting from the surgical intervention. Possible surgical complications such as pain, swelling, and fever and the risks arising from the therapy including nausea, vomiting, or drug intolerance were explained to all patients.

For the duration of the study, all patients were assisted by the surgical team in the event of any kind of postoperative problem, such as infection, uncontrolled pain, fever, or other complications due to the procedures, if necessary.

### 2.5. Outcomes

Immediately after surgery, details of each operation were documented, together with the total duration of surgery. The amount of postoperative pain was the primary outcome variable measured. This allowed the patient to describe their discomfort more objectively. The intensity of the primary pain variable was recorded using a 10-cm VAS, ranging from 0 (no pain) to 10 (maximum pain). Each subject was invited to register their perceived pain at 30 min, 2, 6, 12, 24, 48 h, 7 and 10 days after the completion of surgery. At this stage, any additional analgesics or other drugs taken by each study participant were recorded.

The second outcome analyzed was the appearance of postoperative swelling. For the analysis of this outcome, pre- and postoperative values (obtained at each follow-up session, i.e. at 24, 72 h, 5, 7 and 10 days) of the different facial measurements were compared, as described previously [12]: mandibular angle to tragus (distance MA-Tr), mandibular angle to external corner of the eye (distance MA-ECE), mandibular angle to nasal border (distance MA-NB), mandibular angle to labial commissure (distance MA-LC), and mandibular angle to soft pogonion (distance MA-SP).

For the clinical analysis of possible trismus, the maximum degree of mouth opening was measured. This was done at baseline, 24, 72 h, 5, 7 and 10 days after surgery using a calibrated sliding caliper (TheraBite range of motion scales). The measurement was made by calculating the difference between the baseline value and that obtained at each follow-up session.

### 2.6. Statistical Analysis

The Kolmogorov–Smirnov test revealed that the data examined were not normally distributed. Therefore, a non-parametric approach was taken for the analysis of the data. The Kruskal–Wallis test was applied to compare pain scores, facial distances, and maximum mouth opening among the three groups at each observation time point. The Mann–Whitney test was applied for two-by-two comparisons. The Friedman test was applied for the comparison of the measurements (pain scores, facial distances, and maximum mouth opening values) at the different observation time points within each group. The Wilcoxon test was used to perform two-by-two comparisons between time observations, for each follow-up session. For the primary outcome, Bonferroni’s correction was applied for the multiple comparisons, for which the significant alpha level 0.050 has to be divided by the number of possible comparisons between 8 time points, compared two-by-two. The “adjusted” significance level for this analysis was equal to 0.050/28 = 0.0018. The receiver operating characteristic (ROC) curve was realized in order to show an overall area under the curve for the three treatments, providing an additional level of resolution into their efficacy over the entire trial period. Thus, for each treatment, the area under the curve (AUC) was calculated with a relative 95% confidence interval (CI) and significance. The significance of the P-value was set at 0.05. The statistical analyses were performed using SPSS 17.0 for Windows statistical software (SPSS Inc., Chicago, IL, USA).

## 3. Results

All enrolled patients completed the study without any postoperative complications. The mean age of the 91 patients (44 male, 47 female) found to be eligible for the study was 29.6 ± 2.8 years. Twenty-nine patients (15 male, 14 female) were randomly allocated to the placebo group, 31 patients (15 male, 16 female) to the Flurbiprofen group, and 31 patients (14 male, 17 female) to the Lornoxicam group. Postoperative healing was uneventful in all patients and without any adverse events such as infections or abscesses during follow-up.

The peak postoperative pain score was seen at 12 h in the placebo, 12 h in the Flurbiprofen group, and 6 h in the Lornoxicam group (Table 1). 

**Figure 2:**
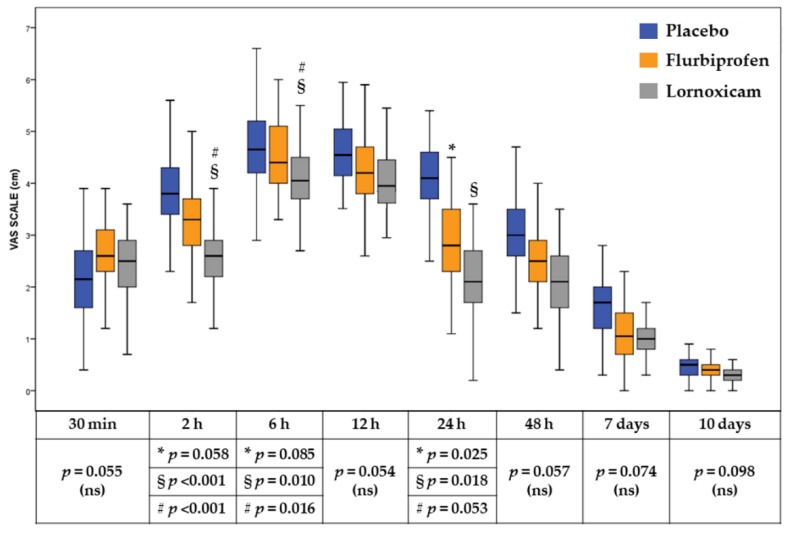
Median and inter-quartile range of VAS (visual analogue scale) pain intensity score and significance of comparisons between study groups. *, placebo vs Flurbiprofen; §, placebo vs Lornoxicam; #, Flurbiprofen vs Lornoxicam; ns, not significant.

Moreover, a further comparison in the median and interquartile VAS score between the groups at each follow up session is represented in Table 2 and in Figure 2. At 2 h following surgery, the median VAS score was significantly lower in the Lornoxicam group compared with the Flurbiprofen (*p* < 0.001) and with the placebo (*p* < 0.001) group. At 6 h following surgery, the median VAS score was significantly lower in the Lornoxicam group compared with the Flurbiprofen (*p* = 0.016) and with the placebo group (*p* = 0.01). At 24 h following surgery, compared with the placebo group, the median VAS score was significantly lower in the Flurbiprofen (*p* = 0.025) and Lornoxicam group (*p* = 0.018) (Figure 2).

The differences between time points of the VAS score are represented in Table 3. All treatment groups presented significant differences between the eight follow-up time points (*p* = 0.000). More specifically, the placebo group presented VAS score significantly at every time point, excluding when the VAS score values at 2 h were compared to the values obtained at 24 h (*p* = 0.0049) and when the VAS score at 6 h was compared to the values at 12 h (*p* = 0.453). The Flurbiprofen group improved the VAS score significantly at every time point, excluding when the VAS score values at 30 min were compared to the values obtained at 24 h (*p* = 0.0743), 48 h (*p* = 0.954) and when the VAS score at 6 h was compared to the values at 12 h (*p* = 0.0022). The Lornoxicam group improved the VAS score significantly at every time point, excluding when the VAS score values at 30 min were compared to the values obtained at 2 h (*p* = 0.0730), 24 h (*p* = 0.0048) and at 48 h (*p* = 0.066), when the VAS score at 6 h was compared to the values at 12 h (*p* = 0.1503) and when VAS score values at 24 h were compared to the values at 48 h (*p* = 0.7068).

**Figure 3:**
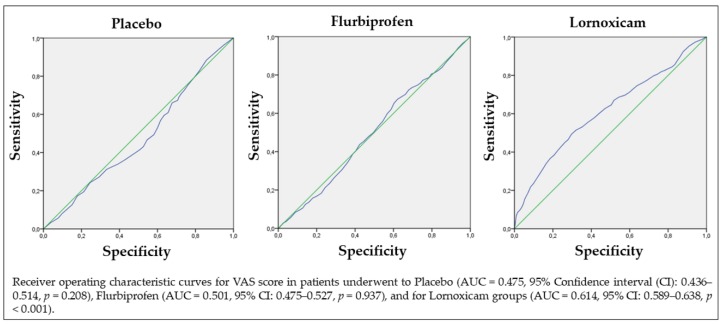
Overall area under the curve (AUC) for the three treatments. *p*-value < 0.001.

Furthermore, regarding the overall area under the curve (AUC), for the placebo, an AUC = 0.475 (Confidence Interval (CI) 95% = 0.436–0.514), *p* = 0.208 was obtained, which was not significant. Similarly, for Flurbiprofen an AUC = 0.501 (CI 95% = 0.475–0.527), *p* = 0.937 was obtained, which was not significant; for Lornoxicam, instead, a higher AUC = 0.614 (CI 95% = 0.589–0.638) with a *p*-value < 0.001 was obtained (Figure 3).

There were no significant differences between the groups regarding the quantity of dental anesthetic used: placebo group, 2.6 ± 0.6; Flurbiprofen group, 2.2 ± 0.4; and Lornoxicam group, 2.4 ± 1.3 (*p* = 0.344). The mean duration of surgery was similar in the three groups: 24.56 ± 5.31 min for the placebo group, 23.19 ± 5.24 min for the Flurbiprofen group, and 24.41 ± 5.18 min for the Lornoxicam group (*p* = 0.166). The osteotomy and tooth sectioning was performed without intraoperative accidents or complications in all enrolled patients.

Measurements of the facial distances, recorded pre- and postoperatively to determine the degree of swelling in the study groups, did not differ between the three groups at any of the observation time points (*p* > 0.05) (Table 4). Moreover, the maximum mouth opening values did not differ significantly between the treatment groups at any of the observation time points (*p* > 0.05) (Table 3). 

However, the comparison of maximum mouth opening between time points in each of the study groups highlighted statistically significant differences when the baseline values were compared to postoperative values (placebo group at 24 h, *p* < 0.001 and 7 days, *p* = 0.003); the Flurbiprofen group at 24 h (*p* < 0.001) and 5 days (*p* = 0.007); and the Lornoxicam group at 24 h (*p* < 0.001) 72 h (*p* = 0.007), and 5 days (*p* = 0.007) (Table 4). Moreover, in the Lornoxicam group, a significant difference in mean maximum mouth opening was demonstrated postoperatively when the value at 5 days was compared to the value at 24 h postoperatively (*p* = 0.007) (Table 5).

## 4. Discussion

The objective of this study was to evaluate the effectiveness of Flurbiprofen and Lornoxicam in the prevention of postoperative discomfort after mandibular third molar surgery. More specifically, it sought to assess the efficacy of Flurbiprofen and Lornoxicam in the management of postoperative pain, facial swelling, and mouth opening compared to placebo. Treatment with Lornoxicam resulted in a favorable significant decrease in the appearance of postoperative pain compared to Flurbiprofen and placebo during the first phases of postoperative healing following the surgical avulsion of the impacted mandibular third molar.

During recent years, several NSAIDs have been demonstrated to be effective among anti-inflammatory drugs for the postoperative management of pain following the extraction of impacted third molars [19]. However, different studies have demonstrated adverse drug effects in patients on NSAIDs following their administration [20,21]. Olmedo et al. [22] reported that 37.3% of the patients who required adjunctive therapy with Ketorolac or Ketoprofen following third molar surgery presented adverse events such as drowsiness (10.7%), pyrosis (10.3%), and gastric lesions (8%).

Among the NSAIDs, Lornoxicam has been demonstrated to be efficacious for dental pain management [14,23]. Mojsa et al. [13] reported favorable effects using a single pre-emptive dose of Lornoxicam (16 mg) for the analgesic therapy in third molar surgery. Based on these preliminary results, the present study was performed to analyze the effects of Lornoxicam compared to Flurbiprofen and placebo, as postsurgical therapy following impacted mandibular third molars. The results of this study showed that the postoperative administration of Lornoxicam significantly reduced postoperative pain in the period of the first 24 h compared to Flurbiprofen and placebo.

Pektas et al. [24] evaluated the preventive analgesic effectiveness of Diflunisal compared to Lornoxicam. In that study, the authors observed that Diflunisal and Lornoxicam provided similar results at 2, 4, 6, 12 and 24 h postoperatively. Moreover, Lustenberger et al. [25] found that when Lornoxicam was used to manage postoperative discomfort following third molar surgery, its efficacy with regard to the management of early stage acute pain was comparable to that of ibuprofen.

In the present study, a 16 mg dose of Lornoxicam exhibited a substantial analgesic effect during the first postoperative 24 h compared with 200 mg Flurbiprofen and with placebo. The group of patients who received Lornoxicam therapy showed a peak in postoperative pain at 6 h following the intervention; pain values then decreased continuously over the subsequent follow-up sessions. In contrast, the peak pain score occurred at 12 h in the placebo group and 12 h in the Flurbiprofen group and scores remained higher up, in all groups, to 48 h following surgery. Moreover, the Lornoxicam group presented significantly lower median pain scores at 2 h (*p* < 0.001) and at 6 h (*p* = 0.016) compared to Flurbiprofen and at 2 h (*p* < 0.001), 6 h (*p* = 0.01), and at 24 h (*p* = 0.018) after surgery compared with placebo, suggesting a better analgesic efficacy of Lornoxicam compared to the other treatments during the first 24 h of healing after surgery (Figure 2). Moreover, compared to the other treatments, the treatment with Lornoxicam presented a higher AUC (*p* < 0.001) which provided additional proof of resolution of the efficacy of Lornoxicam over the entire of the study period.

The results of this study are in agreement with those of other authors who have shown that Lornoxicam at an intravenous dose of 8-mg 25 min before surgery is effective in the short-term reduction (already at 2 h) of the appearance of postoperative pain in patients undergoing dental procedures, oral malignancies, or third molar surgery [25,26,27,28].

With regard to swelling values, all groups presented comparable results. The swelling can be explained by the inflammatory and edema responses that occur as a result of surgical trauma. This mechanism occurs mainly through the production of prostaglandins and cyclooxygenases, which are synthesized following arachidonic acid release from the cell membrane of cells at the surgical site [24]. Despite our results, some studies have, however, previously shown that Lornoxicam represents one of the best mediators, similar to other NSAIDs, for the reduction of the release of arachidonic acid, resulting in a clinical reduction in swelling [24,29,30].

The assessment of trismus, measured by comparing the maximum mouth opening values obtained at baseline to those obtained at each follow-up session, showed a significant decrease in the Lornoxicam group at 24, 72 h and 5 days after surgery. Similarly to the present study, Norholt et al [30], who performed a study in which patients took morphine, and Tuzuner Oncul et al. [29], who performed a study in which patients took diclofenac, demonstrated that the use of NSAIDs determined a significant improvement of trismus reduction following third molar surgery. However, in our study, treatment with Lornoxicam and Flurbiprofen determined a significant reduction in the maximum mouth opening values only up to 5 days after surgery, while at 10 days, the results were comparable between all treatments.

Positive effects of Lornoxicam include its association with fewer episodes of pyrosis and upper and lower gastrointestinal lesions compared to other NSAIDs, also in patients with periodontal disease or who underwent oral anticoagulant therapy [21,31,32,33,34].

## 5. Conclusions

During the last few decades, different drugs have been proposed with the aim of reducing postoperative discomfort by helping to reduce pain and swelling following surgery without causing adverse effects. Identifying even more effective drugs or combinations of drugs for pain management following third molar surgery, with the purpose of discovering treatment strategies other than NSAIDs or corticosteroids, should be encouraged.

This study suggests that Lornoxicam used as postoperative therapy after third molar surgery shows favorable effects in the first phases of perioperative pain management compared to Flurbiprofen and placebo. However, there were no differences between treatments for the reduction of postoperative swelling and trismus.

The results of this preliminary study are encouraging; however, further research is required to provide a better understanding of the potential benefits of Lornoxicam in postoperative therapy following impacted third molar surgery.

## Figures and Tables

**Table 1:** Maximum peak visual analogue scale (VAS) pain intensity score value in the study groups.

Period	Placebo (*n* = 29)	Flurbiprofen (*n* = 31)	Lornoxicam (*n* = 31)
30 min	4	4	4
2 h	5	5	4
6 h	6	6	6
12 h	7	7	5
24 h	5	4	3
48 h	5	4	4
7 days	3	2	2
10 days	1	1	1

VAS: visual analogue scale.

**Table 2:** Median VAS pain intensity score in the study groups. Results are represented as the median ± standard error of the mean (SEM).

Period	Study Groups (Median ± SEM)		
	Placebo (*n* = 29)	Flurbiprofen (*n* = 31)	Lornoxicam (*n* = 31)
30 min	2.1 ± 0.08	2.6 ± 0.07	2.4 ± 0.07
2 h	3.8 ± 0.07	3.3 ± 0.08	2.6 ± 0.06
6 h	4.4 ± 0.06	4.8 ± 0.07	3.9 ± 0.07
12 h	4.5 ± 0.06	3.7 ± 0.08	3.8 ± 0.06
24 h	4.1 ± 0.05	2.8 ± 0.08	1.9 ± 0.08
48 h	3 ± 0.07	2.5 ± 0.06	2 ± 0.07
7 days	1.6 ± 0.04	1.1 ± 0.06	0.9 ± 0.04
10 days	0.4 ± 0.02	0.4 ± 0.02	0.3 ± 0.01

**Table 3:** *p*-values of global and two-by-two comparison between 8 time points performed by Friedman test and Wilcoxon test, respectively. Adjusted α level = 0.0018. ns, not significant.

Comparisons Between Time-Point Follow-Up	Placebo (*n* = 29)	Flurbiprofen (*n* = 31)	Lornoxicam (*n* = 31)
Comparison between 8 follow-up time points	0.000	0.000	0.000
30 min vs 2 h	0.000	0.000	0.0730 (ns)
30 min vs 6 h	0.000	0.000	0.000
30 min vs 12 h	0.000	0.000	0.000
30 min vs 24 h	0.000	0.0743 (ns)	0.0048 (ns)
30 min vs 48 h	0.000	0.0954 (ns)	0.0066 (ns)
30 min vs 7 days	0.000	0.000	0.000
30 min vs 10 days	0.000	0.000	0.000
2 h vs 6 h	0.000	0.000	0.000
2 h vs 12 h	0.000	0.000	0.000
2 h vs 24 h	0.0049 (ns)	0.002	0.000
2 h vs 48 h	0.000	0.000	0.000
2 h vs 7 days	0.000	0.000	0.000
2 h vs 10 days	0.000	0.000	0.000
6 h vs 12 h	0.453 (ns)	0.0022 (ns)	0.1503 (ns)
6 h vs 24 h	0.000	0.000	0.000
6 h vs 48 h	0.000	0.000	0.000
6 h vs 7 days	0.000	0.000	0.000
6 h vs 10 days	0.000	0.000	0.000
12 h vs 24 h	0.000	0.000	0.000
12 h vs 48 h	0.000	0.000	0.000
12 h vs 7 days	0.000	0.000	0.000
12 h vs 10 days	0.000	0.000	0.000
24 h vs 48 h	0.000	0.0094	0.7068 (ns)
24 h vs 7 days	0.000	0.000	0.000
24 h vs 10 days	0.000	0.000	0.000
48 h vs 7 days	0.000	0.000	0.000
48 h vs 10 days	0.000	0.000	0.000
7 days vs 10 days	0.000	0.000	0.000

ns: not significant.

**Table 4:** Differences in the facial distance measurements recorded pre- and postoperatively in the study groups. Data are expressed as the mean ± standard deviation (SD). MA, mandibular angle; Tr, tragus; ECE, external corner of the eye; NB, nasal border; LC, labial commissure; SP, soft pogonion.

Distances	Differences in cm (mean ± SD)			*p*-value
	Placebo (*n* = 29)	Flurbiprofen (*n* = 31)	Lornoxicam (*n* = 31)	
Baseline				
MA-Tr	5.1 ± 0.5	5.2 ± 0.5	5.1 ± 0.5	0.789
MA-ECE	9.3 ± 0.6	9.1 ± 0.4	9.3 ± 0.7	0.855
MA-NB	9.4 ± 0.5	9.5 ± 0.2	9.7 ± 0.5	0.544
MA-LC	7.8 ± 0.7	7.7 ± 0.2	8.8 ± 0.6	0.657
MA-SP	9.3 ± 0.9	9.6 ± 0.9	9.3 ± 0.3	0.848
24 h				
MA-Tr	5.3 ± 0.6	5.4 ± 0.6	5.4 ± 0.5	0.748
MA-ECE	9.5 ± 0.7	9.2 ± 0.7	9.3 ± 0.8	0.596
MA-NB	9.9 ± 0.7	10.1 ± 0.5	10.2 ± 0.9	0.647
MA-LC	8.8 ± 0.5	8.8 ± 0.6	8.7 ± 0.5	0.558
MA-SP	10.1 ± 0.4	9.4 ± 0.5	8.7 ± 0.7	0.896
72 h				
MA-Tr	6.2 ± 0.8	6.5 ± 0.6	6.5 ± 0.6	0.557
MA-ECE	10.1 ± 0.3	9.8 ± 0.5	10.3 ± 0.7	0.742
MA-NB	10.9 ± 0.3	10.7 ± 0.2	11.2 ± 0.9	0.716
MA-LC	9.6 ± 0.3	9.6 ± 0.6	9.6 ± 0.5	0.638
MA-SP	11.1 ± 0.5	11.4 ± 0.5	10.4 ± 0.6	0.604
5 days				
MA-Tr	5.5 ± 0.5	5.8 ± 0.5	5.9 ± 0.3	0.664
MA-ECE	10.3 ± 0.4	9.9 ± 0.5	9.8 ± 0.5	0.785
MA-NB	11.1 ± 0.3	9.8 ± 0.6	10.5 ± 0.6	0.496
MA-LC	8.9 ± 0.2	8.8 ± 0.7	8.2 ± 0.7	0.557
MA-SP	10.2 ± 0.8	10.5 ± 0.5	10.8 ± 0.8	0.898
7 days				
MA-Tr	5.3 ± 0.3	5.5 ± 0.8	5.5 ± 0.4	0.456
MA-ECE	9.7 ± 0.8	9.4 ± 0.5	9.4 ± 0.7	0.558
MA-NB	10.1 ± 0.2	10.1 ± 0.7	10.6 ± 0.4	0.571
MA-LC	8.8 ± 0.8	8.3 ± 0.5	8.9 ± 0.8	0.865
MA-SP	9.4 ± 0.8	9.7 ± 0.9	9.5 ± 0.5	0.749
10 days				
MA-Tr	5.5 ± 0.3	5.5 ± 0.2	5.2 ± 0.3	0.985
MA-ECE	9.5 ± 0.3	9.7 ± 0.4	9.3 ± 0.5	0.539
MA-NB	10.2 ± 0.2	9.7 ± 0.3	9.7 ± 0.5	0.789
MA-LC	8.8 ± 0.4	8.5 ± 0.5	8.5 ± 0.5	0.369
MA-SP	9.8 ± 0.4	9.1 ± 0.5	9.2 ± 0.7	0.596

MA-Tr: mandibular angle to tragus (distance MA-Tr); MA-ECE: mandibular angle to external corner of the eye (distance MA-ECE); MA-NB: mandibular angle to nasal border (distance MA-NB); MA-LC: mandibular angle to labial commissure (distance MA-LC); MA-SP: mandibular angle to soft pogonion (distance MA-SP).

**Table 5:** Comparison of maximum mouth opening recorded pre- and postoperatively in the study groups. Data are expressed as the mean ± standard deviation (SD). Significance of comparisons between time points and baseline assessments: *p < 0.001; † *p* = 0.003; ‡ *p* < 0.001; § *p* = 0.007; ‖ *p* < 0.001; ¶ *p* = 0.007; # *p* = 0.007.

Period	Study Groups (mean ± SD)			*p*-value
	Placebo (*n* = 29)	Flurbiprofen (*n* = 31)	Lornoxicam (*n* = 31)	
0 h	41.2 ± 3.5	40.7 ± 2.4	41.3 ± 2.8	0.597
24 h	32.4 ± 3.6 *	33.1 ± 3.4 ‡	32.5 ± 2.6 ‖	0.647
72 h	35.8 ± 3.3	35.9 ± 3.6	37.8 ± 2.7 ¶	0.679
5 days	36.8 ± 2.5 †	36.2 ± 3.4 §	36.8 ± 3.3 #	0.771
7 days	36.5 ± 3.5	35.9 ± 2.2	36.2 ± 3.2	0.854
10 days	39.7 ± 2.6	42.5 ± 3.1	40.9 ± 2.7	0.691

## Supplementary Materials

The following are available online at https://www.mdpi.com/2077-0383/8/3/325/s1, Table S1: CONSORT 2010 checklist of information to include when reporting a randomized trial.
